# Statin treatment for primary and secondary prevention in elderly patients—a cross-sectional study in Stockholm, Sweden

**DOI:** 10.1007/s00228-024-03724-3

**Published:** 2024-07-16

**Authors:** Björn Wettermark, Camelia Kalantaripour, Tomas Forslund, Paul Hjemdahl

**Affiliations:** 1 Department of Pharmacy, Faculty of Pharmacy, Uppsala University, Box 580, 751 23 Uppsala, Sweden; 2 Academic Primary Health Care Centre, Stockholm Region, Stockholm, Sweden; 3 Department of Medicine Solna, Clinical Epidemiology Unit, Karolinska Institute and Clinical Pharmacology, Karolinska University Hospital, Stockholm, Sweden

**Keywords:** Drug utilization, Statins, Elderly, Hyperlipidemia, Cardiovascular prevention

## Abstract

**Background:**

Age is a major risk factor for atherosclerotic cardiovascular disease (CVD) and death, but there has been a debate about benefit-risk of statin treatment in the elderly with limited evidence on benefits for primary prevention, while there is strong evidence for its use in secondary prevention.

**Aim:**

The aim of this study was to provide an overview of statin utilization in primary and secondary prevention for patients 75–84 years and ≥ 85 years in the Swedish capital Region Stockholm in 2019.

**Methods:**

This is a cross-sectional study based on the regional healthcare database VAL containing all diagnoses and dispensed prescription drugs for all 174,950 inhabitants ≥ 75 years old in the Stockholm Region. Prevalence and incidence were analyzed by sex, age, cardiovascular risk, substance, and the intensity of treatment.

**Results:**

A total of 35% of all individuals above the age of 75 in the region were treated with statins in 2019. The overall incidence in this age group was 31 patients per 1000 inhabitants. Men, individuals 75–84 compared to ≥ 85 years of age, and those with higher cardiovascular risk were treated to a greater extent. Simvastatin was used primarily by prevalent users and atorvastatin by incident users. The majority was treated with moderate-intensity dosages and fewer women received high intensity treatment.

**Conclusions:**

Statins are widely prescribed in the elderly. Physicians seem to consider individual cardiovascular risk when deciding to initiate statin treatment for elderly patients, but here may still be some undertreatment among high-risk patients (especially women and elderly 85 + years) and some overtreatment among patients with low-risk for CVD.

## Introduction

Aging populations and increased survival rates from myocardial infarction (MI) and stroke have increased the need for cardiovascular prevention [1]. There is strong evidence that statins reduce cardiovascular morbidity and mortality [2, 3]. However, most studies have included younger patients, and the number of patients above the age of 75 years has been limited. Current guidelines may not be so well suited for elderly patients with cardiovascular risk and many other concomitant diseases and drugs [3–5], and the benefit-risk ratio for statin treatment in the elderly has been a subject of debate [6].

There is solid evidence of beneficial effects of statin treatment for secondary prevention in elderly patients. A meta-analysis (CTTC) from 2019 showed a significant risk reduction for cardiovascular events among elderly ≥ 75 years with established CVD treated with statins [7]. Statins are thus indicated for elderly patients with high cardiovascular risk, including those with a prior myocardial infarction (MI), ischemic heart disease (IHD), stroke/TIA, or peripheral atherosclerotic disease (PAD). Consequently, current European guidelines recommend statins for secondary prevention in the elderly [3, 4].

The evidence for a favorable benefit-risk ratio for primary prevention with statins in the elderly is less robust and mainly based on subgroup analyses of small RCTs [8–11] or observational studies. Some observational studies have shown risk reductions of the same magnitude or even greater than for younger patients [12–16], but there are also observational studies showing limited benefit of statin treatment for primary prevention in elderly individuals with and without diabetes [17, 18]. In a meta-analysis of 28 RCTs published in 2019, no significant cardiovascular risk reduction was shown for primary prevention among the 8% elderly patients > 75 years (RR 0.92; 99% KI 0.73–1.16) [7]. The significant trend towards smaller proportional risk reductions with increasing age remained even after exclusion of 4 studies on heart failure and kidney dialysis (where statins have not been shown to be effective) [7].

Current guidelines suggest that statin treatment in the elderly should be based on individual benefit-risk assessments, taking age, risk for adverse drug reactions (ADRs), comorbidities, polypharmacy, drug-drug interactions, pharmacodynamics, and pharmacokinetics as well as patient preferences into account.

Statins are in general well tolerated and double blind RCTs have shown low frequencies of ADRs which indicates that tolerability problems partly reflect patient expectations [2, 19]. Muscular symptoms are the most common ADRs and, e.g., in the PRIMO observational study 10.5% of all patients treated with statins reported myalgia [20]. However, there is a substantial nocebo-effect, and according to the SAMSON study, similar symptom intensity was experienced among participants during statin treatment compared to placebo treatment [21]. Furthermore, no difference was found between statin and placebo in terms of symptom intensity at start and extent of symptom relief at discontinuation [21]. This indicates that many patients experience side effects linked to taking tablets rather than the tablet containing a statin. Other ADRs include the development of diabetes, but this risk is very low [2]. There is no evidence that statin have any negative impact on cognition [22], and according to the PALM study, statins have similar tolerability in older and younger patients [23].

The debate has been intense among professional societies and the general public on the value of treating elderly patients with statins. Limited evidence of the beneficial effect in primary prevention, potential problems of poor medication adherence, drug–drug interactions, and side effects that could negatively impact quality of life have fueled this debate. There is limited knowledge on how this is reflected in the prescribing patterns of physicians and to what extent elderly with different cardiovascular risk profiles are treated with statins today. The aim of this study was to provide an overview of the use of statins in primary and secondary prevention for elderly (≥ 75 years) and very old (≥ 85 years) patients in the Stockholm Region in 2019. We specifically assessed if there were any differences in incidence and prevalence of statin treatment, and choice of drug and dose by age, sex, and cardiovascular risk profile of the patients.

## Methods

### Study design and population

This was a cross-sectional study based on registry data on dispensed prescription drugs and recorded diagnoses in primary and secondary care for all individuals over the age of 75 in the Swedish capital region of Stockholm.

### Data sources

Data were collected from the administrative healthcare database VAL held by the regional health authority in Stockholm [24]. VAL contains information about all hospitalizations and specialist ambulatory care consultations in the region since 1993 (with more than 99% coverage), and all primary care consultations since 2003 (with more than 85% coverage of diagnoses). The database also contains demographic information on patient age, sex, migration, and death. For each healthcare contact, the VAL database contains a record of the provider unit, an encrypted patient identification number, age and sex, the type and length of the stay, and up to ten diagnoses. Since 1997, diagnoses are coded according to the WHO’s International Classification of Diseases, 10th edition (ICD-10). Since July 2010, information on dispensed prescription drugs is also included in the database. These data originate from the same data source as the Swedish Prescribed Drug Register with a population coverage of over 99% [25]. The Anatomical Therapeutic Chemical (ATC) classification system is used to classify different drugs [26].

For this study, we extracted all dispensed prescriptions of statins (ATC C10AA) to residents in the Stockholm Region at any pharmacy in the country during 2018 and 2019, respectively, and linked this with information on diagnoses reported in primary care, specialist ambulatory care and inpatient care during the years 2015–2019, using the unique patient identifiers for each patient [27]. Data on dispensed prescriptions included age and sex of the patient, dispensing date, information about the dispensed drug (substance, ATC-code, formulation, and strength), and the specialty of the prescriber.

The diagnoses included in the study were MI (ICD I21, I22, I24.1, and I25.2), IHD (I20, I24 excl. I24.1, and I25 excl. I25.2–4), stroke/TIA (G45, G46, I63-I66, and I69.3–4), PAD (I70, I71, I73.9, I74, and K55), diabetes mellitus (E10, E11), and hypertension (I10).

### Data analyses

Analyses were conducted for incident and prevalent statin users. The first prescription dispensed in 2019 was selected to identify substance and strength in all analyses. Prevalence was calculated as the proportion of all elderly living in the Region (January 1, 2019) who purchased at least one prescription of a statin during 2019 and presented as a number of patients/1000 inhabitants. To calculate incidence, a washout period of one year was applied, i.e., patients without any dispensing in 2018. Incidence was defined as the proportion of all people in the Region who claimed their first prescription and presented as a number of patients/1000 person-years.

Age was classified into two groups: 75–84 vs. 85 and above. Sex was classified as men and women. A diagnosis hierarchy developed by Wallach Kildemoes et al. was applied to manage cardiovascular comorbidity in the analyses of cardiovascular risk [28]. In this approach, each individual is classified based on the highest ranked diagnoses reported during the previous five years. The diagnosis hierarchy applies the following ranking of diagnoses: MI (1), IHD (2), stroke/TIA (3), PAD (4), diabetes (5), hypertension (6), and none of these diagnoses (7).

Four different statins were available on the Swedish market at the time of the study: atorvastatin (C10AA05), simvastatin (C10AA01), rosuvastatin (C10AA07), and pravastatin (C10AA03). Their dose intensities were classified as low, moderate, or high (Table 1).

**Table 1:** Definition of statin intensity for elderly [12, 29]

Statin	ATC	Low intensity	Moderate intensity	High intensity
Atorvastatin	C10AA05	–	10–20 mg	40–80 mg
Pravastatin	C10AA03	20 mg	40 mg	–
Rosuvastatin	C10AA07	–	5–10 mg	20–40 mg
Simvastatin	C10AA01	10 mg	20–40 mg	80 mg*

^*^The highest dose of simvastatin is not recommended due to less favorable risk–benefit

### Statistical analyses

Descriptive data are presented as numbers and proportions of patients and as means and ranges. Data management and analyses were performed using SAS 9.4 [SAS Institute, Cary, NC] and Microsoft Excel 2016.

## Results

During 2019, the region had 128,627 inhabitants aged 75**–**84 and 46,323 inhabitants over the age of 85, corresponding to 5 and 2%, respectively, of the total population (all ages) in the region (Table 2). There were more women than men in both age groups (55% among 75–84 years and 66% among 85 + years of age). The overall cardiovascular risk was higher among men compared to women in both age groups (Table 2).

**Table 2:** Individuals with a history of cardiovascular disease in the Stockholm Region during 2015–2019 per age and sex

	75–84 years				85 + years			
	Men (*n* = 58,418)		Women (*n* = 70 209)		Men (*n* = 15,800)		Women (*n* = 30,523)	
	*N*	(%)	*N*	(%)	*N*	(%)	*N*	(%)
**MI**	6 026	10	3 007	4	2 310	15	2 487	8
**IHD**	8 595	15	4 894	7	3 226	20	3 880	13
**Stroke/TIA**	5 867	10	5 459	8	2 512	16	4 119	13
**PAD**	3 999	7	2 913	4	1 386	9	1 756	6
**Diabetes mellitus**	12 970	22	10 763	15	3 049	19	4 595	15
**Hypertension**	37 102	64	44 394	63	11 113	70	22 765	75
***None of the above***	*16,772*	*29*	*22 332*	*32*	*3 287*	*21*	*6 063*	*20*

Individuals with many concomitant diagnoses are presented more than once

*MI* myocardial infarction, *IHD* ischemic heart disease, *PAD* peripheral artery disease

### Prevalence and incidence of statin treatment

**Fig. 1:**
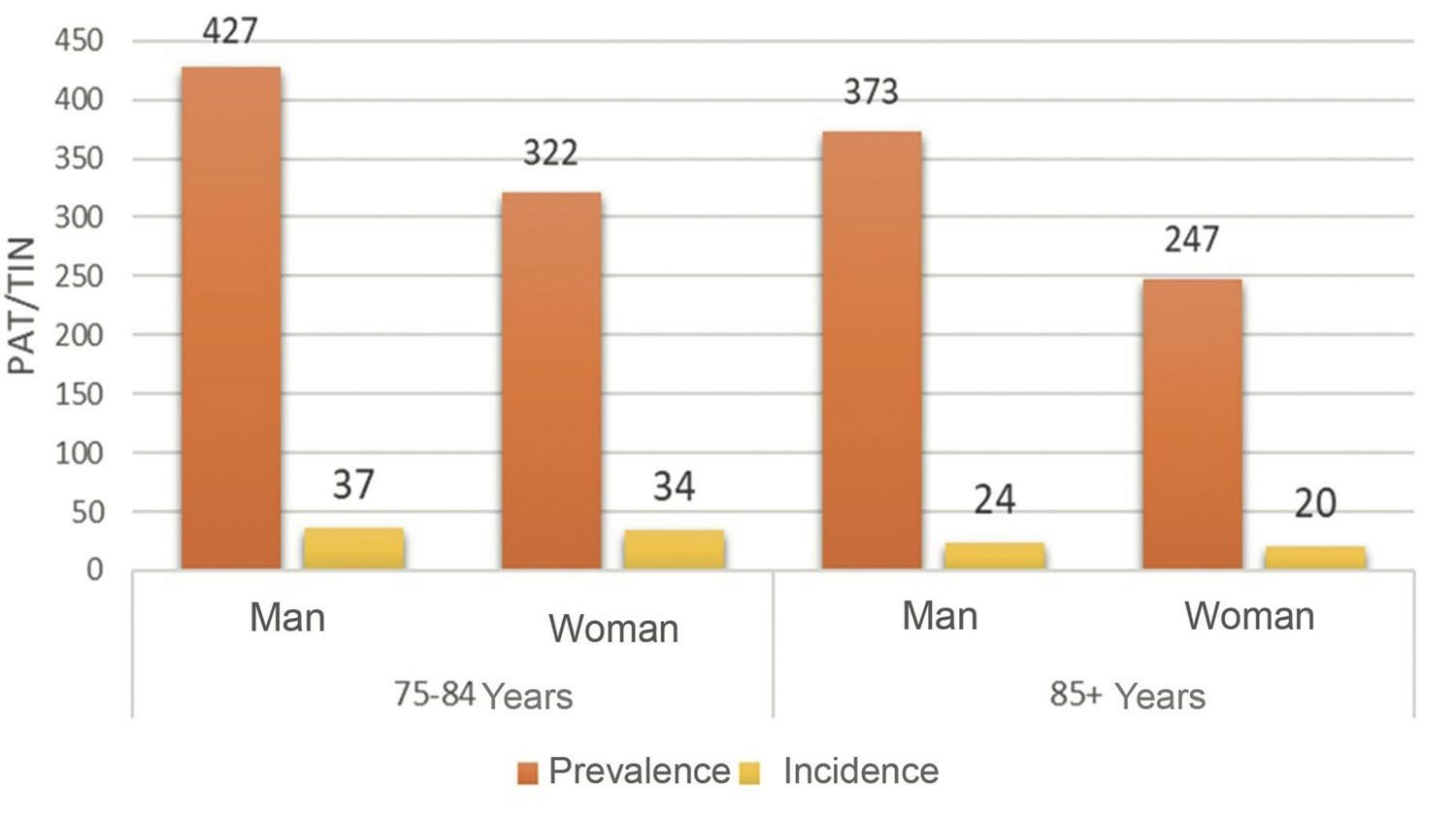
Prevalence and incidence of statin treatment (patients per thousand inhabitants; PAT/TIN) among the elderly by sex and age group in the Stockholm Region 2019

A total of 35% of all elderly in the region were treated with statins in 2019. The lowest proportion was observed among women over the age of 85 with 25% (247 patients/1000 inhabitants) and the highest among men 75–84 years of age with 43% (427 patients/1000 inhabitants) (Fig. 1). The overall incidence was 31 elderly patients per 1000 inhabitants, ranging between 20 and 37 per 1000 inhabitants. Thus, 3% of the total elderly population or around one tenth of the prevalent users were initiated during 2019 (Fig. 1). More men were dispensed and initiated on statins in both age groups.

**Fig. 2:**
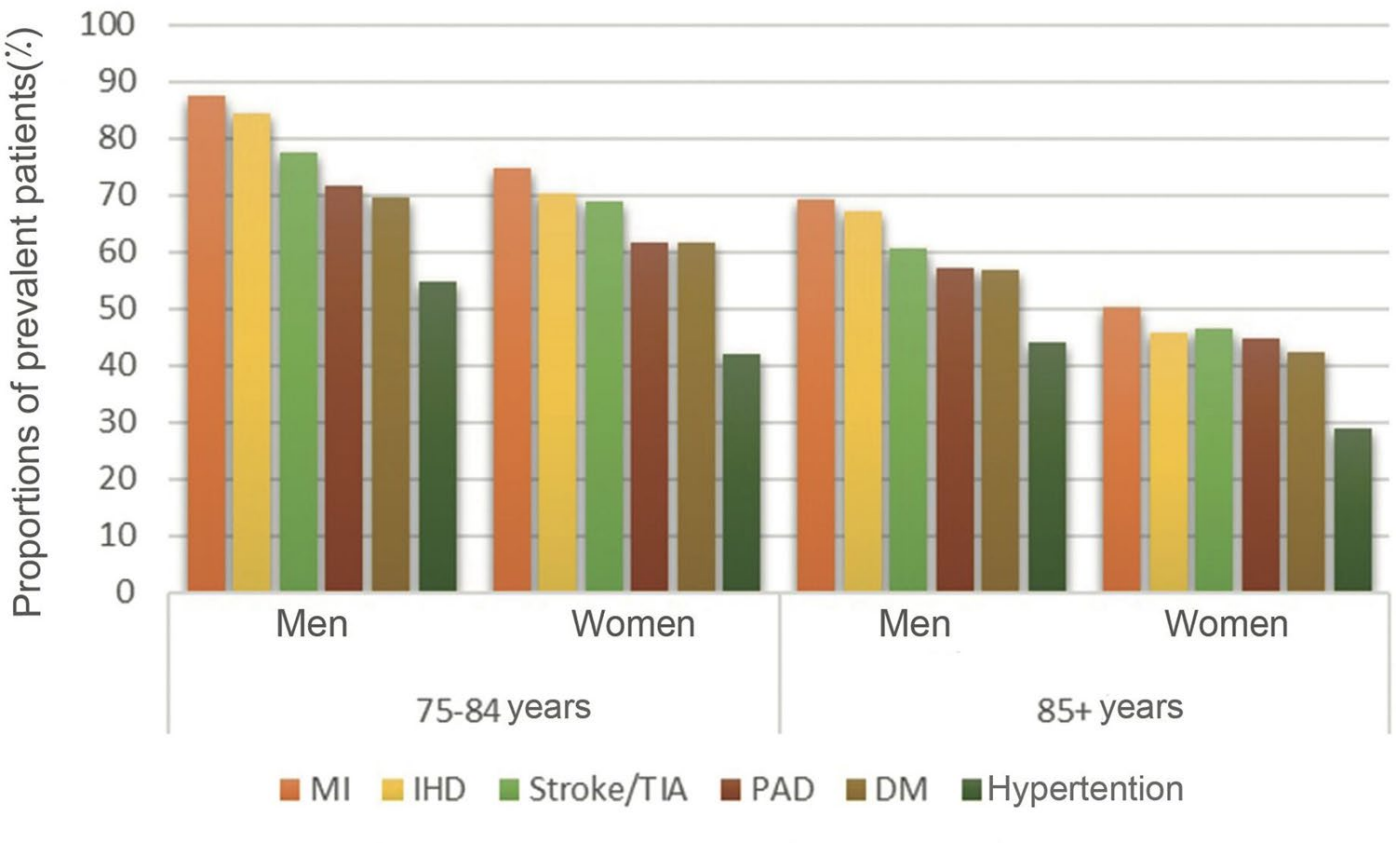
Proportions of elderly individuals in Stockholm dispensed statins during 2019 by age, sex and previous history of cardiovascular disease diagnosed between 2015 and 2019. MI = myocardial infarction, IHD = ischemic heart disease, PAD = peripheral artery disease, DM = diabetes mellitus

Among those 75–84 years of age with an identified cardiovascular risk, the utilization of statins was highest among those who had a history of MI and lowest among those with hypertension (Fig. 2). One tenth (9.8%) of the elderly without a previously registered cardiovascular diagnosis received statin treatment in 2019. The proportions were 11 and 10% among men and women aged 75–84 and 9 and 6% among men and women older than 85, respectively.

### Cardiovascular risk among elderly patients treated with statins

The largest proportion of all women 75–84 years of age who were dispensed statins during 2019 (34%) had only hypertension as a cardiovascular risk factor. Among men of the same age, 21% had only hypertension while an equally high proportion had a history of MI. Among those aged 85 + , hypertension alone was still most common (28%) among women, while a history of MI (alone or in combination with other risk factors) was most common (27%) among men (Table 3).

**Table 3:** Cardiovascular risk among all elderly in the Stockholm Region who were dispensed (prevalence) or newly initiated on (incidence) statins during 2019 by age and sex

Diagnosis	Proportion prevalent patients (%)				Proportion incident patients (%)			
	75–84 + år		85 + år		75–84 år		85 + år	
	Men	Women	Men	Women	Men	Women	Men	Women
1 MI	21	10	27	17	16	9	26	19
2 IHD	14	9	17	13	10	8	10	9
3 Stroke/TIA	13	14	17	19	20	21	27	33
4 PAD	5	5	5	5	7	6	7	8
**Total (secondary prevention)**	**53**	**38**	**66**	**54**	**53**	**44**	**70**	**69**
5 Diabetes mellitus	18	19	12	13	16	16	11	7
6 Hypertension	21	34	17	28	22	29	14	19
7 None of the diagnoses above	8	9	5	5	9	11	5	5
**Total (primary prevention)**	**47**	**62**	**34**	**46**	**47**	**56**	**30**	**31**

In total 53% of all men and 38% of all women aged 75–84 years received statins for secondary prevention. Corresponding figures for those older than 85 were 66% and 54% of all men and women, respectively. The proportion treated for secondary prevention was slightly higher among patients initiated on treatment (Table 3).

### Choice of statin and dose intensity

**Fig. 3:**
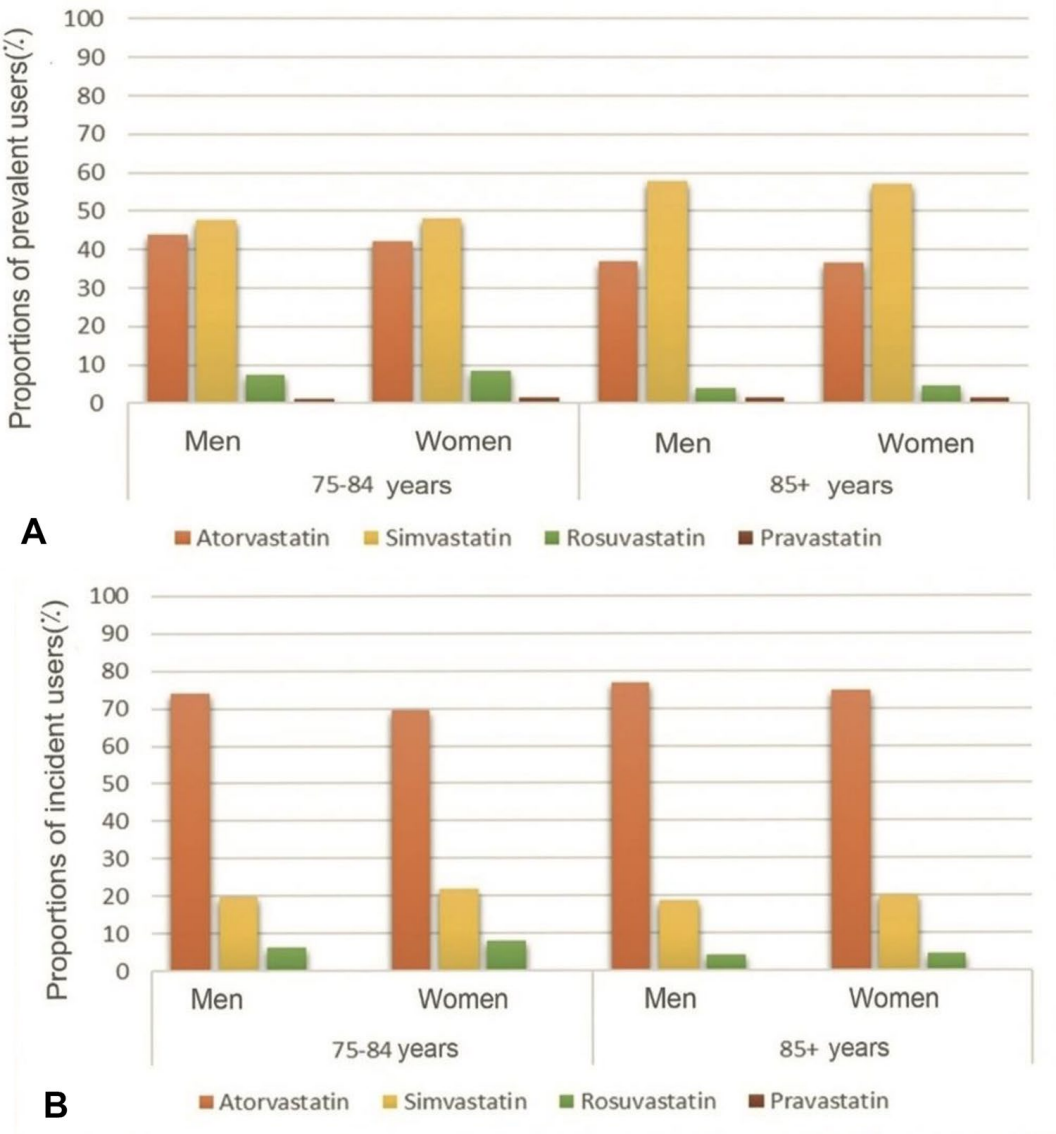
Choice of statins for treatment of elderly in Region Stockholm by age and sex during 2019. **A** All elderly patients (prevalent). **B** Elderly patients initiated on treatment (incident)

Simvastatin was the most common statin among prevalent users in both sex and age groups (Fig. 3A) whereas atorvastatin was most common among incident users (Fig. 3B). Simvastatin was more common among the oldest than among those 75–84, but there were no sex differences in the choice of statins.

**Fig. 4:**
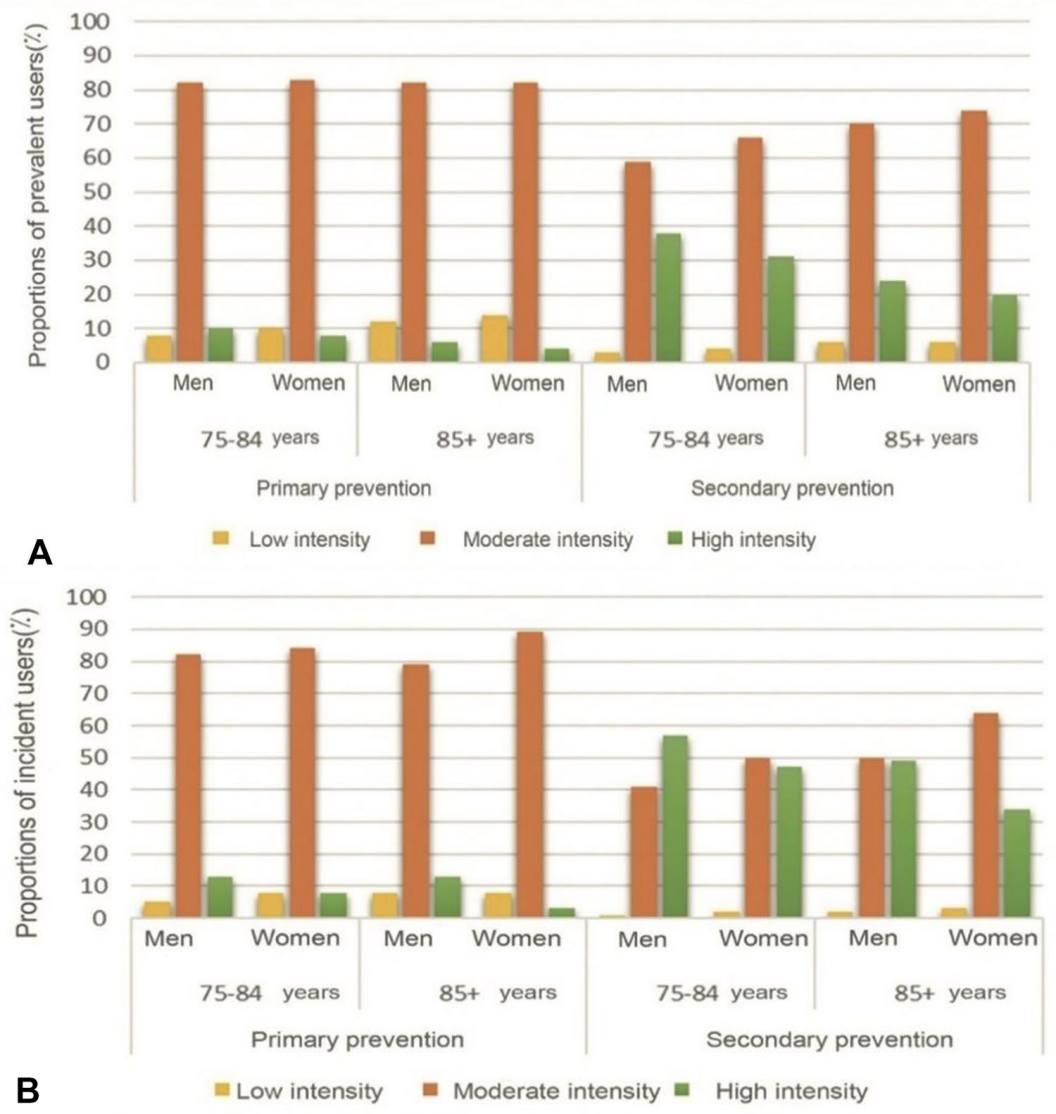
Dose intensity of statins treatment among elderly patients in the Stockholm Region during 2019 by age, sex and primary vs. secondary prevention. **A** All elderly patients (prevalent). **B** Elderly patients initiated on treatment (incident)

Most elderly patients of both sexes received statins with moderate intensity, but the proportion treated with high intensity for secondary prevention was higher among men (Fig. 4A). Low intensity treatment was more common in primary than in secondary prevention for both age groups, while the opposite was observed for high intensity treatment in both age groups (Fig. 4A).

Moderate intensity treatment was also most common among newly initiated patients in most patient groups except for men aged 75–84 treated for secondary prevention where a slightly higher proportion received high intensity treatment (Fig. 4B). Overall, low intensity was more commonly used in primary prevention and high intensity for secondary prevention, regardless of age and sex.

## Discussion

In this large cross-sectional study including all inhabitants over the age of 75 years in the Swedish capital region, we found 35% to be treated and 3% to be newly initiated on statin treatment in 2019. Men, individuals < 85 years and those with higher cardiovascular risk were treated to a higher extent. Simvastatin was prescribed primarily to prevalent users and atorvastatin to incident users. A majority were treated with moderate-intensity dosages and fewer women received high intensity treatment.

The prevalence shows treatment in relation to cardiovascular risk categories which reflects guidelines that have been in place for a long time, while the incidence reflects how physicians are currently reasoning around risk–benefit when initiating treatment for elderly patients. It is not surprising that statin treatment is common among the elderly given that atherosclerosis and other cardiovascular risk factors such as diabetes, hypertension, chronic kidney disease and dyslipidemia increase by age [5, 30]. There are also several studies showing that the benefit of treating elderly patients with statins is similar to that of treating younger patients [7, 31, 32]. Relatively few elderly patients (3%) were initiated on statins in our study, but many high-risk patients were probably already treated.

We found that more men than women received treatment in all cardiovascular risk groups. Since large RCTs have shown similar beneficial effects of statin treatment in men and women, major gender differences should not be expected (CTC 2019). However, similar gender differences in utilization have been found in other studies, despite higher cholesterol levels among the women [33–36]. Several reasons for these inequities have been suggested. There is some evidence that cardiovascular disease is underdiagnosed in women [37]. Furthermore, in a meta‐analysis including more than 1.8 million elderly statin users in 13 countries, female sex was associated with increased nonadherence [38]. The latter may be attributable to more perceived adverse effects in women or limited motivation. More studies are needed to investigate reasons behind these gender differences.

Statin treatment was less common among the oldest, and few people over 85 years of age were initiated on treatment. This is in line with findings from other studies [39, 40]. It may reflect either that physicians consider the benefit-risk balance to be less favorable or that the oldest patients discontinue their treatment to a larger extent, but the latter seems less likely [38]. Many of the oldest (85 + years) are vulnerable with many other concomitant diseases and drugs that may interact with statins and increase the risk for adverse events [41]. Their life expectancy is less than for those 75–84 of age and, consequently, the benefits of treatment may be less pronounced [1]. Furthermore, results from a randomized study indicated that discontinuation of statin treatment during the last year of life may improve quality of life [42]. The sparsity of evidence calls for more studies on the effect and safety of statin treatment in the oldest patients [43] as well as qualitative studies investigating attitudes and beliefs about statins among physicians and patients [44, 45].

We found larger proportions of men and women with established CVD to be treated than those who received it for primary prevention (44–78% vs 16–39%), showing that statins are mainly prescribed to those with the largest potential benefit. Our findings indicate better adherence to guidelines than in other studies. A Canadian study showed that only 12% of patients aged 75–84 years and 6% of elderly 85 + without CVD received statins while 34% and 21%, respectively, with an indication for secondary prevention were treated [46]. It is impossible to determine what would be the optimal proportion treated at a population level without access to data on other risk factors such as lipids, smoking and BMI, as well as data on medication adherence in clinical practice. Our findings indicate that there is undertreatment of elderly patients with established CVD, while this is less certain for patients in primary prevention. The proportions of men and women with no risk marker for CVD who received statins in our study were rather small (6–11%). There may be several reasons why they received treatment, such as hypercholesterolemia without established CVD or a strong heredity for CVD. It is also important to acknowledge that even though we collected diagnoses from the entire healthcare system during five years, some patients may have comorbidities not being recorded as ICD-10 diagnoses. However, we cannot with the current data exclude that there is some overtreatment of low-risk patients with no clear indication for statin treatment. This was shown in a Finnish study where 11% of all patients ≥ 75 years of age with low risk received statin treatment [47].

Simvastatin was the most commonly prescribed statin among prevalent patients whereas atorvastatin was the most common choice for patients who were newly initiated. This is not surprising since simvastatin was recommended as first line drug during many years, due well-documented effects and a lower price as a generic drug compared to the other statins. While simvastatin lost its patent in 2002, atorvastatin and rosuvastatin were not generic until 2012 and 2016, respectively. Before the patent expiry for atorvastatin, regional guidelines recommended simvastatin and the Swedish reimbursement agency TLV only reimbursed atorvastatin and rosuvastatin for patients not reaching treatment targets with simvastatin [48, 49]. Thus, many elderly patients have probably been treated with simvastatin for many years without any need to change the therapy.

Moderate intensity dosages were most commonly used in the present elderly cohorts, in line with current recommendations. Older patients are more vulnerable to ADRs because of age-related changes in pharmacokinetics and pharmacodynamics [50]. Furthermore, many elderly are concomitantly treated with other drugs that may interact with statins and increase the risk of ADRs [41]. Our findings are in concordance with the American PALM study showing that fewer elderly received high intensity treatment for secondary prevention (Nanna et al. 2018). The benefit of treatment to low LDL-targets in the elderly may also be questioned as there are both studies that support high intensity treatment in the elderly [51] and that show limited additional benefit [52].

### Strengths and limitations

This study included all elderly inhabitants in an entire Swedish region. Data were collected from a regional data database of high quality with data on diagnoses and healthcare consultations from primary care, specialist ambulatory care and inpatient hospital care for all inhabitants in the region. Drug utilization patterns were assessed using complete data on all dispensed prescription drugs in the country regardless of reimbursement status or where in the country the patients claimed their prescriptions. We chose a five-year period for the identification of diagnoses as all patents do not have annual appointments with healthcare and diagnosis reporting may be incomplete at some visits. This was based on experience from previous research on patterns of diagnoses by us and others [24, 53–56].

We also acknowledge some limitations. Data on dispensed drugs may differ both from what is prescribed, i.e., the intention of the treating physician, and what is actually ingested by the patients. Statins that are purchased abroad are not included in the register but this is not common, and elderly patients may receive statin therapy without prescriptions when they are hospitalized. There might also have been some misclassification in our estimate of incidence based on a wash-out period of one year to identify new users. Furthermore, we only assessed the first prescription being dispensed during 2019 and previous studies have shown that many patients discontinue their treatment early during the therapy [56]. However, this may vary between settings and in a study of secondary prevention with statins in Stockholm we recently found primary non-compliance (not filling a prescription) in ≈ 20% of the patients but non-persistence was very uncommon once the treatment had been initiated [57]. The selection of the first prescription may have underestimated the use of high intensity treatment since some patients may be initiated on lower doses than the target dose used for long-term treatment. There may also be some misclassification in the reporting of diagnoses as previous studies using secondary databases have shown problems with over and under-reporting of diagnoses [58]. It is unlikely that we have overestimated the cardiovascular risk in our study. We used a five-year time window including all caregivers and diagnoses of MI and stroke and this has been shown to have a high validity [59]. There is, however, a potential that we underestimated the risk for some patients who had no consultations with reported diagnoses during this time window or who were treated abroad or at the very few private clinics not reporting data. Finally, we acknowledge that we only assessed prescribing patterns in relation to registered diagnoses since we had no data on other risk factors such as LDL cholesterol, heredity, renal function, smoking, alcohol, BMI, and physical activity.

## Conclusion

This study showed that more than one third of all elderly, aged ≥ 75 years in the Swedish capital region of Stockholm, are treated with statins. The adherence to guidelines is rather high with most patients at high cardiovascular risk receiving evidence-based statin treatment with appropriate doses. Still, it is likely that there is undertreatment among high-risk patients (especially those with TIA/stroke, PAD, women, and the very old, 85 + years) and some overtreatment among patients with low-risk for CVD. Physicians seem to consider the patient’s cardiovascular risk when deciding to initiate statin treatment for the elderly.

## Data Availability

The pseudonymized patient-level data collected from regional registers are not allowed to share publicly due to confidentiality reasons; however, upon reasonable request, additional analyses can be conducted after contact with the corresponding author.
